# Closing the Numeracy Gap in Medication Safety: Impact of a *safeMedicate* Intervention in Undergraduate Medical Education

**DOI:** 10.1002/prp2.70204

**Published:** 2025-11-30

**Authors:** Soban Sadiq, Susan Driver, Manfred Gschwandtner

**Affiliations:** ^1^ Kent and Medway Medical School University of Kent Canterbury UK

**Keywords:** calculation, essential modules, numeracy, patient safety, *safeMedicate*

## Abstract

Medication errors, often linked to inadequate numeracy skills, pose significant risks to patient safety. To address this, Kent and Medway Medical School (KMMS) became the first UK medical school to integrate *safeMedicate*, a validated e‐learning platform, into its Year 1 undergraduate medical curriculum. This study aimed to evaluate its impact on student engagement, numeracy competence, and confidence. The entire cohort of 111 first‐year medical students (2024 intake) was introduced to the *safeMedicate* Essential Skills module within the Year 1 module titled Professional Development and Person‐Centred Practice. Engagement was assessed via platform analytics (logins, time, completion), numeracy competence through a formative online test, and perceptions via an anonymous survey. Engagement was high, with students averaging 9.1 logins and 124.2 min on the platform. Completion rates were near universal (95%). The average test score was 85.4%, with 75% of students achieving ≥ 85%. Competency analysis showed strong performance in conceptual, calculation, and technical measurement skills. Survey responses indicated that 89% found *safeMedicate* helpful for test preparation and 83% reported increased confidence in numeracy. Students valued the clarity, usability, and practice‐based learning approach. Early integration of *safeMedicate* demonstrated improved engagement, numeracy performance, and student confidence. Although limited to one institution and formative assessment, findings support continued use of structured digital tools to strengthen medication safety education. Embedding *safeMedicate* into undergraduate curricula may reduce prescribing errors and better prepare future doctors for safe clinical practice.

## Graphical abstract

Integration of the *safeMedicate* platform into Year 1 undergraduate medical curriculum significantly improved student engagement, numeracy competence, and confidence in medication safety. High completion rates and strong test performance demonstrate its value as an effective digital tool for strengthening essential prescribing skills.

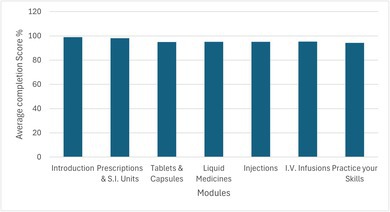

**Abbreviations**
BNF: British National Formulary
KMMS: Kent and Medway Medical School
OSCE: Objective Structured Clinical Examination
PDPCP 1: Professional Development and Person Centred Practice (Year 1)
PSA: Prescribing Safety Assessment

## 1 Introduction

Medication errors represent a significant threat to patient safety within healthcare systems globally. A primary contributing factor to these errors is inadequate clinical numeracy skills among healthcare professionals, particularly in the critical area of drug dosage calculation [1]. Medication and prescription errors represent a significant patient safety concern in the UK. According to a 2018 report commissioned by the Department of Health and Social Care (DHSC), an estimated 237 million medication errors occur annually in England across all stages of medication use including prescribing, dispensing, administration, and monitoring. Of these, approximately 66 million are potentially clinically significant, and more than 1700 deaths annually may be directly linked to medication errors. Prescribing errors are the most frequent, accounting for nearly 50% of all medication errors in primary care. Common issues include incorrect dosages, drug interactions, and misdocumentation [2]. The ability to accurately calculate and administer medication is a foundational competency for all healthcare students and practitioners [3]. Recognizing this imperative, e‐learning platforms have emerged as valuable tools to support the development and assessment of these essential skills [4].

The *safeMedicate* platform has emerged as a widely adopted, evidence‐based tool in nursing education, aimed at developing essential medication dosage calculation skills and fostering clinical safety among nursing students. *SafeMedicate* provides interactive, self‐paced learning modules designed to identify skill deficits and offer targeted remediation. The platform employs authentic competence models, diagnostic feedback, and constructivist learning principles to support real‐world skill development [5, 6]. Its use has expanded globally, with over 330 000 learners across healthcare systems benefiting from its structured approach to medication safety education [5].

Studies across the UK and internationally demonstrate *safeMedicate*'s effectiveness in improving medication dosage calculation skills and assessment accuracy among healthcare students and professionals. For example Hutton et al. [7] validated the *safeMedicate* simulation model by showing a high correlation between OSCE‐based and computer‐delivered assessment outcomes. It was determined that integrating a web‐based authentic assessment environment with additional evaluation of technical measurement interpretation in a practical or simulated setting, using a benchmark and a profession‐validated criterion‐referenced rubric, constitutes an innovative, effective, valid, and reliable method for assessing the safe administration of medications [8, 9, 10]. Students who use *safeMedicate* show notable progress in developing both their understanding and calculation skills for solving medication dosage problems [10, 11]. Research on the pedagogy underpinning *safeMedicate* emphasizes that authentic learning environments effectively engage and accommodate all types of cognitive learning styles, unlike traditional lecture‐based teaching methods [12].

Kent and Medway Medical School (KMMS) is the first medical school in the UK to formally incorporate the *safeMedicate* platform into its undergraduate medical curriculum, aiming to enhance students' numeracy competence in clinical practice. This study details the first implementation of *safeMedicate* within the Year 1 curriculum at KMMS, which began in September 2024. The intervention aimed to address previously poor numeracy outcomes and enhance medication safety competence through a highly structured and interactive learning experience. By examining student engagement, performance, and perceptions, this study contributes to a growing evidence base on the role of digital tools in strengthening clinical numeracy in undergraduate medical education.

## 2 Methods

This study employed a descriptive approach to assess the integration of *safeMedicate* within the Year 1 undergraduate medical curriculum. As this intervention formed part of the routine medical curriculum, and because the survey was conducted as part of the standard module evaluation process, the project was classified as a curricular service evaluation using anonymized educational data. Therefore, in accordance with KMMS policy, formal ethics approval and informed consent were not required. The participants were the entire cohort of 111 first‐year undergraduate medical students enrolled in September 2024 at KMMS. The *safeMedicate* platform was introduced as a formative requirement within the Professional Development and Person‐Centred Practice 1 (PDPCP1) module. Students received an initial introductory session outlining the platform's purpose and requirements. All students were granted access to the platform and advised to complete the “Essential Skills” module. This module comprises seven core sections: Introduction, Prescription, Tablets and Capsules, Liquid Medicines, Injections, Intravenous (IV) Infusion, and Practice Your Skills, each featuring multiple short videos and practice questions. To meet the formative requirements, students were expected to achieve a minimum of 80% engagement across all components of the “Essential Skills” module, including practice exercises. Additionally, students were required to score at least 80% on the formative numeracy test administered through the *safeMedicate* platform. Supplemental drop‐in sessions were also made available to provide further guidance and clarification. Drop‐in sessions were optional academic support sessions delivered by PDPCP1 module faculty (including clinical lecturers), providing small‐group guidance on safeMedicate content, calculation methods, and clarification of module expectations.

## 3 Data Collection

Data were collected directly from the *safeMedicate* platform, including:

- Student login counts.
- Total time spent engaging with the learning material.
- Progress and average completion scores for each component of the ‘Essential Skills’ module.
- Results from the formative numeracy test, including total scores and scores across specific competency components (Conceptual, Calculation, and Technical Measurement Competence).

A short, anonymous survey was conducted after the formative numeracy test to get students' responses. Fifty‐four students completed the survey. It included close‐ended questions with selection options of “agree”, “disagree”, “neutral”, “strongly agree” and “strongly disagree” to assess the platform's perceived helpfulness in improving numeracy skills, preparing for the formative test, and its support for learning and building confidence. One open‐ended question elicited qualitative feedback on the most useful aspects of *safeMedicate* for numeracy learning. Data were collected between April 2025 and May 2025. The survey was administered using Microsoft Forms and the survey questions are provided in the Supporting Information.

## 4 Data Analysis

Quantitative data were analyzed using Microsoft Excel 365. Data analysis was conducted by the first author and reviewed by the other authors. Quantitative data from *safeMedicate* (engagement metrics, module completion scores, and test results) were analyzed to determine averages. Survey data from close‐ended questions were analyzed by calculating the percentage distribution of responses. Free‐text responses to the open‐ended survey question were reviewed and narratively presented.

## 5 Results

### 5.1 *SafeMedicate* Module Engagement and Completion

**FIGURE 1:**
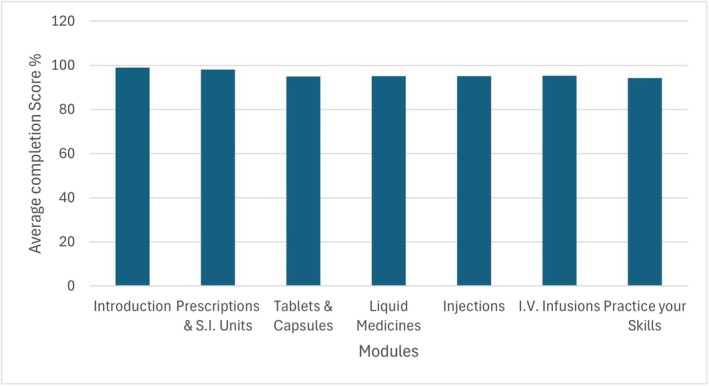
Average completion scores across the different components of the Essential Skills module.

Out of the 111 enrolled students, 95% (*n* = 105) fully engaged with the *safeMedicate* platform. On average, students logged into the platform 9.1 times and spent approximately 124.2 min engaging with the learning material. The data also indicate very high levels of module completion. Most students achieved near‐complete scores in each area, reflecting a strong overall commitment to the learning process (Figure 1).

### 5.2 Formative Numeracy Test Report

**FIGURE 2:**
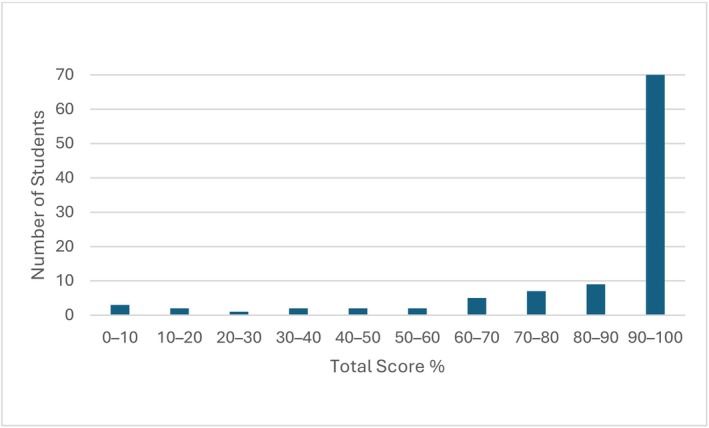
Distribution of total percentage scores.

The formative numeracy test was taken by 103 students. The cohort demonstrated a strong overall performance. The average total score was 54.7 out of a possible 64, which translates to an average percentage score of 85.4%. The median percentage score was notably higher at 96.9%, indicating that half of the students scored above this mark. Most students (75%) scored above 85%. Individual scores ranged from a minimum of 1.6% to a perfect 100% (Figure 2).

### 5.3 Competency Levels

**FIGURE 3:**
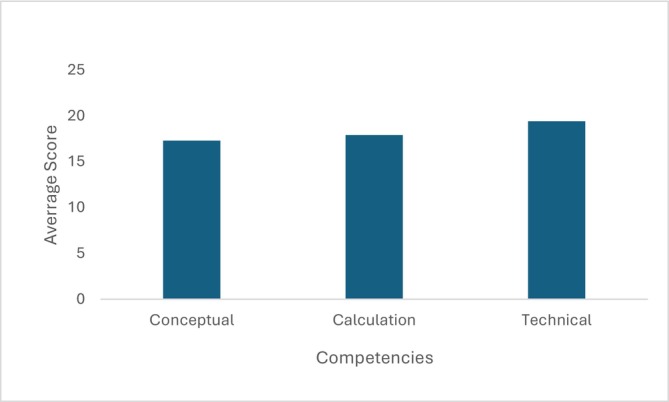
Average score by competency component.

The breakdown of performance by individual competencies reveals additional insights. Conceptual competence refers to understanding units, conversions, and the interpretation of prescriptions; calculation competence relates to performing the arithmetic required for accurate dosage determination; and technical measurement competence involves correctly interpreting measurements from simulated clinical devices. The average score for Conceptual Competence was 17.3 out of 21, and for Calculation Competence it was 17.9 out of 21. Technical Measurement Competence had the highest average score, at 19.4 out of a possible 22 (Figure 3).

### 5.4 Survey Data

This section presents the findings from a survey on the effectiveness of *safeMedicate* modules in supporting numeracy learning and confidence. The survey included four closed‐ended questions and one open‐ended question. A total of 54 students completed the short survey. The figures from the closed‐ended questions are presented in a Supporting Information.

The close‐ended questions data revealed positive perceptions regarding the effectiveness of *safeMedicate*. A significant majority of students (67%) reported an improvement in their numeracy skills after using *safeMedicate*. Excitingly, 89% of students felt that *safeMedicate* was helpful in preparing them for the formative numeracy test. When asked about the overall helpfulness of the *safeMedicate* modules and the “Practice Your Skill” section, 83% of students found them beneficial for supporting their learning and building confidence in numeracy. Furthermore, the “Practice Your Skill” section was largely seen as an effective tool for the application of knowledge, with 63% of students agreeing that it helped solidify what they learned in the modules.

In response to the open‐ended question about the most useful aspects, students consistently emphasized the clarity, simplicity, and overall effectiveness of *safeMedicate*. They particularly valued the clear calculation methods, intuitive platform design, and the practical benefits gained from the practice questions.

## 6 Discussion

The integration of *safeMedicate* into the Year 1 undergraduate medical curriculum has demonstrably improved students' clinical numeracy skills and preparedness for safe prescribing. These findings were reinforced by Hutton et al. [7] who found strong correlations between digital and practical assessments, confirming the reliability of *safeMedicate*'s simulation‐based design. *SafeMedicate* is a web‐based system developed over two decades through translational research, which links fundamental research with clinical application to enhance health outcomes. Its purpose is to aid in learning, integrating, and assessing cognitive skills essential for solving medication dosage problems [5]. This educational tool has undergone thorough evaluation of its effects in professional settings [3, 13, 14]. Unlike teaching methods where students are passive, *safeMedicate* offers an interactive learning environment, aligning with the current trend of active teaching and web‐based educational software [15]. Students (Nursing) using *safeMedicate* in both the UK and USA have shown notable improvements in their understanding and calculation abilities for medication dosage problems [10, 11]. The strong engagement seen at KMMS reflected in high login frequency, module completion rates, and survey feedback supports these trends. Students valued the clarity of instructional videos, the opportunity for repeated practice, and the immediate feedback mechanisms that foster confidence and correct misconceptions in real‐time. These outcomes support findings by Weeks et al. [11] who reported comparable enhancements in the clinical competence of student nurses through the use of *SafeMedicate*. Furthermore, the platform's alignment with British National Formulary (BNF), and its applicability to both foundational and advanced drug calculations, makes it especially relevant to future assessments for example the Prescribing Safety Assessment (PSA).

Taken together, these findings support *safeMedicate*'s role as a pedagogically sound and clinically relevant tool for improving medication safety. Embedding the platform early in the undergraduate medical curriculum equips students with essential numeracy skills that translate into safer prescribing practices and ultimately better patient outcomes.

The high levels of student engagement, characterized by frequent logins and considerable time spent on the platform, combined with near‐perfect module completion scores, suggest that *safeMedicate* effectively captures and maintains student attention. The strong performance in the formative numeracy test, with an average score of 85.4% indicates that students are not only engaging with the material but are also effectively internalizing and applying the concepts. The detailed breakdown by competency further highlights students' proficiency across various aspects of numeracy, including conceptual understanding, calculation accuracy, and technical measurement. The positive feedback from the student survey reinforces the quantitative findings. Students' appreciation for the clarity of calculation methods, the simplicity of the platform, and the value of practice questions directly correlates with the observed improvements in their performance. This suggests that *safeMedicate*'s design and content are well‐aligned with student learning needs and preferences, fostering confidence and reducing anxiety associated with drug calculations. *SafeMedicate*'s alignment with the BNF and its continuous updates ensure that students are equipped with current and relevant knowledge, which is vital for clinical practice. By reinforcing theoretical understanding through practical application, the platform bridges the gap between classroom learning and real‐world healthcare settings [5, 9]. The findings advocate strongly for the continuation and expansion of this intervention. Embedding this training early in the curriculum is not merely about passing a test; it is about instilling fundamental competencies that are critical for patient safety. Sustaining high standards in clinical numeracy will ensure that future cohorts of medical practitioners are well prepared to administer medications safely and accurately, thereby reducing medication errors and improving patient outcomes [1, 16].

## 7 Limitations

This study has several limitations that should be considered when interpreting the findings. Firstly, the study was conducted at a single institution with a specific cohort of Year 1 medical students, which may limit the generalisability of the results to other academic settings and/or student populations. Secondly, the assessment of numeracy skills was primarily based on a formative test conducted via the *safeMedicate* platform. While this provides valuable insights into competence within the platform's context, it may not fully capture performance in real‐world clinical scenarios. Future research could incorporate direct observation of medication administration or objective clinical assessments to provide a more comprehensive evaluation. Thirdly, student engagement data were collected through platform analytics (login counts, time spent, completion scores), which indicate activity but may not fully reflect the depth of learning or understanding. Similarly, the student survey relied on self‐reported perceptions, which can be subject to social desirability bias. The response rate for the survey (54 out of 111 students) was also less than 50%, potentially introducing selection bias and limiting the representativeness of the qualitative feedback. Finally, this study focuses on the immediate impact within the first year of integration; long‐term retention of numeracy skills and their direct correlation with reduced medication errors in future clinical practice warrant further investigation through longitudinal studies.

## 8 Conclusion

This study highlights the effectiveness of integrating the *safeMedicate* e‐learning platform into the Year 1 undergraduate medical curriculum, demonstrating significant improvements in student engagement, clinical numeracy skills, and confidence in medication safety. High engagement rates, strong module completion, and excellent formative test performance underscore the platform's educational value. Positive student feedback further supports its usability and impact. While limited by its single‐centre, formative design, the findings advocate for broader implementation of *safeMedicate* in later years of an undergraduate medical curriculum and its inclusion in summative assessments to sustain clinical numeracy standards. Overall, continued use and investment in such digital tools are vital for enhancing medication safety and preparing competent future healthcare professionals.

## Author Contributions

S.S. and M.G. designed and conducted the study. S.S. did the analysis and M.G. reviewed it. S.S. wrote the manuscript. M.G. and S.D. reviewed the manuscript. S.S. revised it.

## Ethics Statement

As this intervention formed part of the routine medical curriculum, and because the survey was conducted as part of the standard module evaluation process, the project was classified as a curricular service evaluation using anonymised educational data. Therefore, in accordance with KMMS policy, formal ethics approval and informed consent were not required.

## Conflicts of Interest

The authors declare no conflicts of interest.

## Supporting information


**Data S1:** prp270204‐sup‐0001‐DataS1.docx.

## Data Availability

The data that support the findings of this study are available from the corresponding author upon reasonable request.
