# Supplementary material for: Closing the Numeracy Gap in Medication Safety: Impact of a safeMedicate Intervention in Undergraduate Medical Education
Source: Pharmacol Res Perspect. 2025 Nov 30;13(6):e70204. doi: 10.1002/prp2.70204 (PMC12665163; doi:10.1002/prp2.70204)
Supplement: Supplementary file 1 — Data S1: prp270204‐sup‐0001‐DataS1.docx. [file PRP2-13-e70204-s001.docx]

Title:

Closing the Numeracy Gap in Medication Safety: Impact of a *safeMedicate* intervention in Undergraduate Medical Education

Soban Sadiq^1^, Susan Driver^1^ and Manfred Gschwandtner^1^

^1^Kent and Medway Medical School, University of Kent, Canterbury, United Kingdom

Corresponding author: Soban Sadiq ([soban.sadiq@kmms.ac.uk](mailto:soban.sadiq@kmms.ac.uk))

**Supplementary data: The survey was presented to students with 4 close-ended questions and 1 open-ended question**

**Close-ended questions and figures**


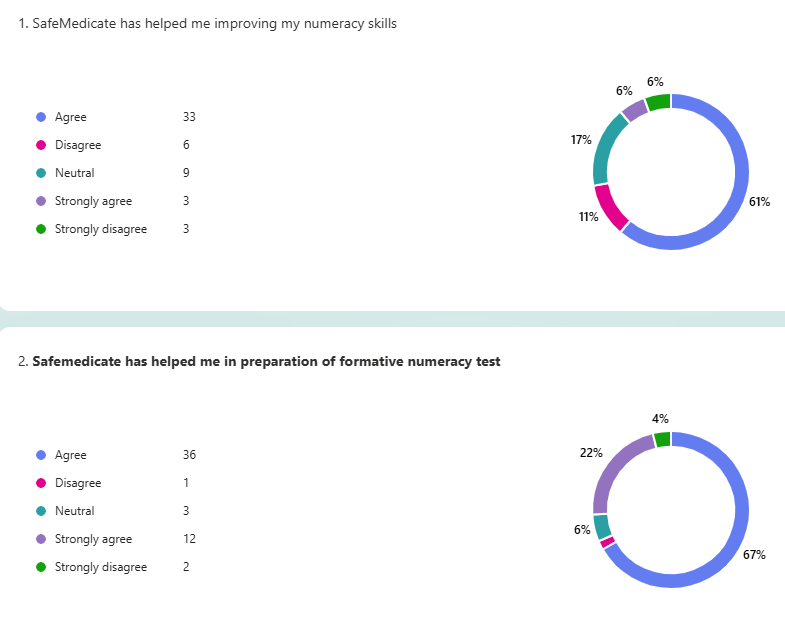


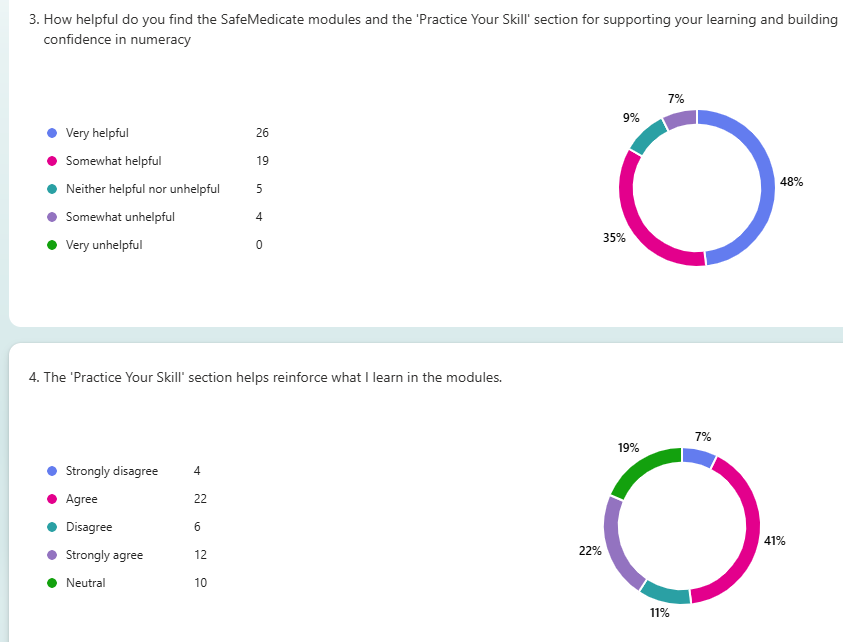


**One open-ended question**

What do you find most useful about using SafeMedicate for learning numeracy?
